# A Case of Uterine Hydatids; Their Expulsion Attended by Alarming Hæmorrhage; Recovery

**Published:** 1846-02

**Authors:** Isaac Edwards

**Affiliations:** Leavenworth, Indiana


### WESTERN JOURNAL

OF

### MEDICINE AND SURGERY.

FEBRUARY, 1846.

**Art. I.**—*A Case of Uterine Hydatids; their expulsion attended by alarming Hæmorrhage; Recovery.* By Isaac Edwards, M.D., of Leavenworth, Indiana.

Mrs. C——, a married lady, æt. 18 years, of moderately full habit, but pale complexion, commenced menstruating in her sixteenth year, and menstruated regularly for nearly twelve months, the catamenia returning about every twenty-seventh day. In about twelve months after the appearance of the catamenial discharge, she took cold, which resulted in a suppression of the menses for two successive periods. Nature, however, at the end of the third month relieved herself; and from that time the lady had her regular periods, the flow being somewhat diminished, up to the second or third month after marriage. Her health during this time appeared to be very good, with the exception of one or two slight attacks of intermittent fever.

Between the second and third month after her marriage, she suffered a suspension of menses, but as this took place while she was apparently in good health, she and her friends were led to believe that she was in the family way. Afterwards, when I interrogated her more closely concerning her feelings about the time of the suppression, she remembered that about the time, or soon after the last uterine discharge, there commenced a burning pain within the pelvic cavity, which lasted, with intervals of freedom from it, for five or six weeks. This pain, however, was so slight that she had never thought proper to mention it to any one, and in fact she had forgotten it herself.

I was called to see the patient August 25th, and found her laboring under, as I thought at the time, menorrhagia, connected with abortion, but, as I afterwards learned, an affection of a different character.

On inquiry I found that she had been troubled with hæmorrhage for some ten days previously; the first symptoms having occurred not long after she had leaped from a horse, which first produced a watery discharge, followed soon afterwards by a considerable flow of blood from the uterus. She complained at the time of my first visit of a considerable headache and pain in the sacral region; the pain shooting out through the hips and around in front of the abdomen, and meeting on the linea alba. She also complained of pain in the left hypochondriac region. At intervals, these symptoms were accompanied by bearing down pains; but as yet these were not very severe. Considerable nausea attended, and sometimes vomiting. A sanguineo-mucous expectoration, slight laryngitis, with an entire loss of appetite, were additional symptoms. Her tongue was covered with a light yellow fur along its middle; mouth dry; considerable thirst. There was a blush on her cheeks between a scarlet and a purple, together with a pale circle around the eyes and mouth. The body was preternaturally warm; with the hands and feet moderately cool; pulse 105, but compressible; bowels constipated.

I prepared the compound rhubarb pills which I ordered her to take to the extent of opening her bowels.

Aug. 26th. Found the patient much better, with the exception of pains in her back and hips, which had become more distressing since the previous day. I now ordered a cantharides plaster over the seat of the pain, to remain on about three hours. I gave her also a gentle tonic, and no other medicine.

27th. Was sent for to-day in haste; and on arriving was informed that twelve hours previously, my patient had again commenced flooding; and that the hæmorrhage had been so great as to induce syncope several times.

On inquiry, I learned that the blister plaster had been allowed to remain on eight hours, and that strangury was the result, together with considerable bearing down pains.

In hope of still being able to avert an abortion, I administered opium and acetate of lead, and used an injection, *per vaginam*, of the lead solution, together with anodyne enemas.

This treatment relieved the strangury in about twenty-five minutes, and also controlled, to a considerable extent, the hæmorrhage; but this persisted in some degree, being sometimes alarming, during the fore part of the night.

About the middle of the night the bearing down pains returned, and increased so rapidly in strength and frequency that all hope of preventing an abortion was given up.

At 2 o'clock I was for the first time permitted to make a thorough examination. Applying my hand to the abdomen just out of cold water, to ascertain if there were any movements of the fœtus, which the lady assured me she had repeatedly felt, I could not perceive any signs of the presence of a child. The uterus, although it was sufficiently large for the seventh month of utero-gestation, did not feel as if it contained a fœtus, the mass, whatever it might be, feeling soft and spongy; this, however, I thought at the time, might be owing to coagula in the uterus. I endeavored in vain to discover a fœtal circulation, and concluded, therefore, that if a fœtus was present, it must be dead.

Next, introducing the index finger of my left hand into the vagina, I ascertained that the os uteri had begun to dilate, and was open to about the size of a dime.

Finding all the means prescribed unavailing, and the patient exhausted from such excessive losses of blood, and concluding that the fœtus must be dead, I immediately determined to bring on labor as speedily as possible.

After communicating my determination to the friends, I gave her the secale cornutum in v. grain doses, repeated every fifteen minutes, together with small doses of the sugar of lead. As soon as these means began to act on the uterus, the hæmorrhage was found to cease; but it still returned again as soon as the effects of the ergot passed off, which appeared to be in about twenty or twenty-five minutes after it was given. I therefore kept it up after one or two intermissions. I gradually increased the dose of the ergot in quantity, but extended the intervals to twenty minutes. By the continued action of the remedy upon the uterus, the hæmorrhage was kept in check.

At half past 6 o'clock, A.M., I made another examination *per vaginam*, and found the os tincæ very much dilated; and protruding through it was a mass feeling so much like a placenta, that I told the woman that it was an after-birth presentation. The dose of ergot was increased to ten grains. About 7 o'clock the hæmorrhage returned more copiously, and I saw that unless there was a speedy delivery, the powers of the patient must succumb. Continuing to give the ergot, I now resorted to a stream of cold water upon the naked abdomen as an auxiliary, in the hope that more effectual uterine contractions might be induced. This I repeated several times, having the skin wiped dry after each application, in order to avoid the depressing effects of continued cold.

This had the desired effect; for immediately after the third dash, the uterus expelled the greater part of its contents. I

was of course very much surprised when I lifted the extruded body from under the sheet, to find that it was not a placenta or a fœtus, but a large mass of *hydatids.* They would have filled a half gallon measure. They were attached to what appeared to be, a blasted placenta.

The parent hydatids were three in number; the middle one being much the largest; the uppermost one when in situ being the next largest. The largest one was about the size and shape of a hen's egg. The largest of the three was the only one that appeared to be organized, and permeated by bloodvessels. It was composed of four different sacs; the innermost one was filled with a yellow serous fluid, not unlike the serum of the blood. The other two parent hydatids were similar to the largest. The larger of these two was composed of four, while the smallest was composed of only three sacs, one within the other. But all were filled with a fluid of the same character.

The parisitic, or secondary hydatids, took their origin from the three parents; the largest primitive one giving off three; the next largest, four; and the smallest, one; making eight in number. They were fastened to their respective parents by small pedicles or footstalks. These again gave off a large number of hydatids of all sizes, from that of a buck-shot up; each one being composed of more laminæ or membranes than one; some two, some three, and some four, but none of a larger number.

After these came away I introduced my index and second finger into the womb, by which I was enabled to detach and bring away the small remnant of the placenta which was left behind. I then gave the patient wine whey and sulphate of quinine, under which she gained strength very fast.

She is now enjoying very good health, but has not since menstruated.

December 31st, 1845.

